# PARG inhibition suppresses osteoclast differentiation through transcriptional activation of an NRF2-dependent pathway

**DOI:** 10.1371/journal.pone.0361101

**Published:** 2026-10-26

**Authors:** Yuka Sasaki, Ryusuke Nakatsuka, Ryoji Taniguchi, Amane Inoue, Mitsuko Masutani, Tadashige Nozaki

**Affiliations:** 1 Department of Pharmacology, School of Dentistry, Osaka Dental University, Hirakata, Osaka, Japan; 2 Department of Molecular and Genomic Biomedicine, Center for Bioinformatics and Molecular Medicine, Nagasaki University Graduate School of Biomedical Sciences, Sakamoto, Nagasaki, Japan; Tokyo University of Agriculture, JAPAN

## Abstract

Poly (ADP-ribose) glycohydrolase (PARG) is the main enzyme responsible for degrading poly (ADP-ribose) (PAR), which is synthesized by PARP family proteins during the poly ADP-ribosylation cycle. We previously reported that the PARG inhibitor PDD00017273 suppressed osteoclast differentiation through PAR accumulation in RANKL-stimulated RAW264 cells. However, the molecular mechanisms of osteoclast differentiation under PARG inhibition remain unclear, and elucidation of the regulatory mechanisms may contribute to the development of novel therapeutic strategies for metabolic bone diseases associated with excessive activation of osteoclasts. In the present study, we investigated the molecular mechanisms whereby PDD00017273 suppresses osteoclast differentiation. RNA-seq and pathway analyses revealed enrichment of oxidative stress- and redox regulation-related pathways, and an ATAC-seq analysis identified enrichment of the NRF2-binding motif in PDD00017273-treated RAW264 cells compared with DMSO-treated control cells. Pharmacological inhibition of NRF2 partially restored the osteoclast formation suppressed by PDD00017273. Under these conditions, the expression levels of NRF2-regulated genes were suppressed, while the expression levels of osteoclast marker genes were upregulated. Taken together, the present findings suggest that PAR accumulation via PARG inhibition suppresses osteoclast differentiation through NRF2 activation, indicating that PAR metabolism plays an important role in osteoclast differentiation.

## Introduction

Bone homeostasis is maintained through a balance between bone resorption by osteoclasts and bone formation by osteoblasts [1,2]. Abnormal bone remodeling arising from excessive activation of osteoclasts can lead to metabolic bone diseases, such as osteoporosis and Paget’s disease of bone, as well as bone metastasis of various cancers [3,4]. Osteoclasts are multinucleated giant cells that were differentiated from hematopoietic stem cells via monocyte–macrophage lineage progenitor cells [5,6]. During osteoclast differentiation, receptor activator of NF-κB ligand (RANKL), produced by osteoblasts, binds to RANK expressed on osteoclast precursor cells, thereby activating downstream signaling pathways, such as the mitogen-activated protein kinase pathway and the NF-κB pathway [7,8]. The resulting signaling induces nuclear factor of activated T-cells, cytoplasmic 1 (NFATc1), a master transcription factor of osteoclast differentiation, and promotes osteoclast differentiation by regulating the expression of relevant genes [7–9].

To date, several osteoclast-targeting therapeutic drugs, including bisphosphonates, the anti-RANKL antibody denosumab, and the selective estrogen receptor modulator raloxifene, have been developed and are currently in clinical use for the treatment of metabolic bone diseases [10,11]. Although these drugs, particularly bisphosphonates and denosumab, show efficacy against metabolic bone diseases, they can also cause serious side effects, such as atypical femoral fractures and medication-related osteonecrosis of the jaw [12,13]. Therefore, it is essential to understand the molecular mechanisms that regulate osteoclast differentiation and to identify potential therapeutic targets for metabolic bone diseases.

Poly ADP-ribosylation (PARylation) is a post-translational modification that adds poly (ADP-ribose) (PAR) to target proteins using β-nicotinamide adenine dinucleotide (NAD^+^) as a substrate [14,15]. The reaction is mainly catalyzed by poly (ADP-ribose) polymerase 1 (PARP1) and PARP2, which are members of the PARP family. Meanwhile, the degradation of PAR polymer is catalyzed by poly (ADP-ribose) glycohydrolase (PARG), the principal PAR-degrading enzyme [15–17]. The PARylation cycle is involved in various biological processes, including DNA repair, cell death, transcriptional regulation, and chromatin remodeling [18–20]. Recent studies have suggested that the PARylation cycle also contributes to the regulation of osteoclast differentiation. PARP1 interacts with the promoter regions of the *Trap* gene and the *Tcirg1* gene, encoding the a3 isoform of the V-ATPase *a* subunit, and acts as a transcriptional repressor to negatively regulate their transcription during osteoclastogenesis [21,22]. PARP1 ADP-ribosylates histone H2B, which negatively regulates NFATc1 expression and osteoclast differentiation [23]. Treatment with the PARP inhibitor olaparib and PARP1/2 deficiency have been shown to promote osteoclast differentiation and cause bone loss in mouse models [24]. Furthermore, we previously reported that PARG inhibition by PDD00017273 leads to PAR accumulation and suppresses osteoclast differentiation [25].

The above findings suggest that the PARylation cycle regulated by PARG and PARP proteins and intracellular PAR accumulation are closely associated with osteoclast differentiation and bone homeostasis. However, while it is becoming clear that PARP enzymes suppress osteoclast differentiation through transcriptional regulation and chromatin modification, the mechanisms by which PARG regulates osteoclast differentiation are not fully understood. Therefore, the present study aimed to elucidate the molecular mechanisms that regulate osteoclast differentiation under PARG dysfunction through a comprehensive analysis of gene expression and chromatin accessibility.

## Materials and methods

### Cell culture and reagents

The murine macrophage RAW264 cell line was obtained from RIKEN Cell Bank (Tsukuba, Japan). The cells were maintained in Minimum Essential Medium (Nacalai Tesque, Kyoto, Japan) supplemented with 10% fetal bovine serum (Gibco, Thermo Fisher Scientific, Tokyo, Japan), 1 × non-essential amino acids solution (Nacalai Tesque), and 1% penicillin-streptomycin (Nacalai Tesque) in a humidified atmosphere containing 5% CO_2_ at 37°C. For differentiation induction, RAW264 cells were seeded at a density of 6.0 × 10^3^ cells/cm^2^ in maintenance medium. After culture for approximately 5 h, the culture medium was replaced with MEM containing 200 ng/mL RANKL (Oriental Yeast, Kyoto, Japan), and the cells were cultured for 0–4 days. The PARG inhibitor PDD00017273 (MedChemExpress, Monmouth Junction, NJ, USA) and the nuclear factor erythroid 2-related factor 2 (NRF2) inhibitor ML385 (MedChemExpress) were dissolved in DMSO and added as needed.

### Tartrate-resistant acid phosphatase (TRAP) staining

To evaluate the osteoclast differentiation abilities, TRAP staining was carried out using a TRACP and ALP Double-Staining Kit (Takara Bio, Shiga, Japan) in accordance with the manufacturer’s instructions. Briefly, cells were washed with phosphate-buffered saline (PBS), fixed with a base solution containing acetone and methanol, and incubated with a substrate solution for acid phosphatase (NABP/FRVLB) supplemented with sodium tartrate at 37°C for 30 min. TRAP-positive multinucleated cells (≥3 nuclei) were counted as osteoclast cells under a light microscope (Olympus, Tokyo, Japan), and representative images were taken using a BZ-X800 microscope (Keyence, Osaka, Japan).

### TRAP activity

To confirm the osteoclast differentiation, TRAP activity was measured using a TRACP & ALP Assay Kit (Takara Bio) in accordance with the manufacturer’s instructions. Briefly, cells were washed with PBS(-) and then solubilized in an extraction solution containing 1% NP-40. The resulting cell lysate was mixed with a reaction substrate solution containing pNPP and sodium tartrate solution, followed by incubation at 37°C. The enzymatic reaction was stopped by the addition of NaOH, and the absorbance at 405 nm was measured using a microplate reader (Infinite F Plex, TECAN, Männedorf, Switzerland).

### Quantitative RT-PCR (qRT-PCR)

Total RNA was extracted from the cells using an RNeasy Mini Kit (Qiagen, Venlo, The Netherlands). Subsequently, cDNA was synthesized from the total RNA using iScript Reverse Transcription Supermix (Bio-Rad, Hercules, CA, USA) in accordance with the manufacturer’s instructions. The obtained cDNA was subjected to qRT-PCR analysis using SYBR Select Master Mix (Thermo Fisher Scientific, Waltham, MA, USA) in a StepOnePlus Real-Time PCR System (Thermo Fisher Scientific). The thermal cycling conditions were as follows: 50°C for 2 min, followed by an initial denaturation step at 95°C for 2 min, and then 40 cycles of denaturation at 95°C for 15 s and annealing/extension at 60°C for 1 min. The target mRNA levels were normalized to the mRNA levels of glyceraldehyde-3-phosphate dehydrogenase (*Gapdh*). The primer sequences for qRT-PCR are listed in Table 1.

**Table 1:** Primer sequences for qRT-PCR.

Target gene	Forward (5’ to 3’)	Reverse (5’ to 3’)
*Trap*	GACAAGAGGTTCCAGGAGACC	GGGCTGGGGAAGTTCCAG
*Ctsk*	GAAGAAGACTCACCAGAAGCAG	TCCAGGTTATGGGCAGAGATT
*Hmox1*	AAGCCGAGAATGCTGAGTTCA	GCCGTGTAGATATGGTACAAGGA
*Nqo1*	AGGATGGGAGGTACTCGAATC	AGGCGTCCTTCCTTATATGCTA
*Slc7a11*	GATTCATGTCCACAAGCACAC	AGAGCATCACCATCGTCAGA
*Gclm*	ACATTGAAGCCCAGGATTGG	CCCCTGCTCTTCACGATGAC
*Gsta2*	GCCTGCCTTTGAAAAGGTGT	ATAGAGAAGAAGTTCCAGCAGGTG
*Nrf2*	GCCCACATTCCCAAACAAGAT	CCAGAGAGCTATTGAGGGACTG
*Keap1*	CAGCAGCGTGGAGAGATATG	GGTTAGTCCCGTCAAAGCCC
*Gapdh*	TGGTGAAGGTCGGTGTGAAC	AGGGGTCGTTGATGGCAACA

### RNA-sequencing (RNA-seq) analysis

RAW264 cells were seeded into 6-well plates at a density of 7.1 × 10^3^ cells/cm^2^ in maintenance medium and cultured for approximately 5 h. The cells were then treated with 1 μM PDD00017273 or DMSO in the presence of 200 ng/mL RANKL for 72 h, followed by extraction of total RNA from the cells. RNA-seq analysis was performed using pooled samples containing equal amounts of material from three independent biological replicates. The RNA quality was assessed by DNA Chip Research Inc. (Tokyo, Japan) using an Agilent Technologies 2100 Bioanalyzer (Agilent Technologies, Santa Clara, CA, USA), and an RNA integrity number of 10 was confirmed. Subsequently, RNA-seq libraries were prepared using a TruSeq Stranded mRNA Library Prep Kit (Illumina, San Diego, CA, USA), and RNA sequencing was performed using a NovaSeq6000 system (Illumina) by DNA Chip Research Inc. The identified sequences were subjected to a pathway analysis by DNA Chip Research Inc. using PathVisioRPC v1.2 (PathVisio 3).

### ATAC-sequencing (ATAC-seq) analysis

The ATAC-seq analysis was performed by DNAFORM Inc. (Kanagawa, Japan). Briefly, RAW264 cells were seeded into 10 cm dish at a density of 6.0 × 10^3^ cells/cm^2^ in maintenance medium, cultured for approximately 5 h, and treated with 1 μM PDD00017273 or DMSO in the presence of 200 ng/mL RANKL for 48 h. Subsequently, the cells were harvested, cryopreserved in BAMBANKER medium (GC Lymphotec, Tokyo, Japan), and shipped to DNAFORM Inc. The ATAC-seq analysis was performed using pooled samples containing equal amounts of material from three independent biological replicates. ATAC-seq libraries were prepared using a standard Tn5 transposase-based protocol. Paired-end sequencing was performed on a NextSeq 2000 system (Illumina). Mapping and peak calling were conducted using the ENCODE ATAC-seq pipeline. The reads were mapped to the mm10 reference sequence using Bowtie2 (ver. 2.3.4.3) [26], and duplicate reads were removed by Picard (ver. 2.20.7) and Samtools (ver. 1.9). Peak calling was performed using MACS2 (ver. 2.2.4) [27] with default parameters. After removal of blacklist regions, peak annotation was conducted using HOMER (ver. 4.9.4) [28] with default settings. Pearson correlation coefficients were calculated based on the genome-wide ATAC-seq signal intensities to assess the similarity between samples. A list of the differential accessible peaks was obtained using DEseq2 (ver. 1.20.0) [29]. Known motifs and de novo consensus motifs in the central 200 bp of the obtained peaks were searched using HOMER with default settings.

### Fractionation of cytoplasmic and nuclear proteins

RAW264 cells were seeded into 10 cm dishes at a density of 9.0 × 10^3^ cells/cm^2^ in maintenance medium, cultured for approximately 4 h, and treated with 1 μM PDD00017273 or DMSO in the presence of 200 ng/mL RANKL for 48 h. Subsequently, the cells were harvested by scraping in PBS, and cytoplasmic and nuclear protein fractions were prepared using NE-PER Nuclear and Cytoplasmic Extraction Reagents (#78833; Thermo Fisher Scientific) in accordance with the manufacturer’s instructions. The samples were stored at −80°C until western blot analysis.

### Western blot analysis

Western blot analysis was performed as described previously [25]. Briefly, whole-cell lysates or cytoplasmic and nuclear protein fractions prepared from RAW264 cells were separated by 5%–20% SDS-polyacrylamide gel electrophoresis and transferred onto PVDF membranes. The following antibodies were used for immunoblotting: anti-β-actin (dilution 1:5000; A2228; Sigma-Aldrich), anti-PAR (dilution 1:2000; #83732; Cell Signaling Technology, Danvers, MA, USA), anti-KEAP1 (dilution 1:1000; #8047; Cell Signaling Technology), anti-NRF2 (dilution 1:1000; #20733; Cell Signaling Technology), anti-α-tubulin (dilution 1:5000; 66031–1-Ig; Proteintech, Tokyo, Japan), and anti-lamin B1 (dilution 1:5000; 12987–1-AP; Proteintech). Immune complexes were detected with horseradish peroxidase-linked secondary antibodies (Cytiva, Tokyo, Japan). Chemiluminescence signals were detected with an Immobilon Western Chemiluminescent HRP substrate (Merck, Branchburg, NJ, USA) and visualized using a ChemiDoc Imaging System (Bio-Rad Laboratories). Image quantification was carried out using Image J software (NIH, Bethesda, MD, USA).

### Statistical analysis

Statistical analyses were performed using GraphPad Prism 10 software (GraphPad Software, San Diego, CA, USA). Data are presented as mean ± standard error (SEM) for values obtained from at least three independent experiments. A value of *p* < 0.05 was considered statistically significant. Comparisons between two groups were performed using Student’s *t*-*t*est, and comparisons among multiple groups were conducted by one-way analysis of variance (ANOVA), followed by Tukey’s HSD post-hoc test. The statistical significance of pathway enrichment was evaluated using permuted *p*-values based on 1,000 permutations.

## Results

### PDD00017273 induces activation of oxidative stress-related pathways during osteoclast differentiation

Previously, we demonstrated that PDD00017273, a PARG inhibitor, suppressed osteoclast differentiation in RANKL-stimulated RAW264 cells via intracellular PAR accumulation [25]. In the present study, treatment with 1 μM PDD00017273 increased the intracellular PAR levels by 2.2-fold compared with DMSO treatment (Fig 1A), supporting the inhibition of PARG enzymatic activity by PDD00017273. PDD00017273 also significantly suppressed osteoclast formation in RAW264 cells at 96 h after RANKL stimulation (Fig 1B, C). To investigate the mechanism by which PARG inhibition suppresses osteoclast differentiation, we comprehensively evaluated the mRNA expression levels by RNA-seq analysis. In the analysis, 466 differentially expressed genes (DEGs) (|log2FC| ≥ 1) were identified between DMSO-treated control cells and PDD00017273-treated cells, comprising 203 upregulated genes and 263 downregulated genes. Notably, several osteoclast differentiation-related genes were downregulated in PDD00017273-treated cells, including *Trap*, *Ctsk*, *Dcstamp*, *Blimp1*, and *Calcr* (Fig 1D). Meanwhile, the expression levels of *Parg*, *Parp1*, and *Parp2* were similar between DMSO-treated control cells and PDD00017273-treated cells. A subsequent pathway analysis indicated that various cellular signaling pathways were altered by PDD00017273 treatment (Fig 1E). Several pathways related to lipid metabolism, such as “eicosanoid synthesis,” “eicosanoid lipid synthesis map,” and “eicosanoid metabolism via lipoxygenases (LOX),” which have been reported to be involved in the promotion of osteoclast differentiation [30,31], were significantly enriched. Several pathways related to antioxidant response and oxidative stress, including “oxidative stress and redox pathway,” “oxidative damage response,” and “transcriptional activation by *Nfe2l2* in response to phytochemicals,” were also enriched.

**Fig 1:**
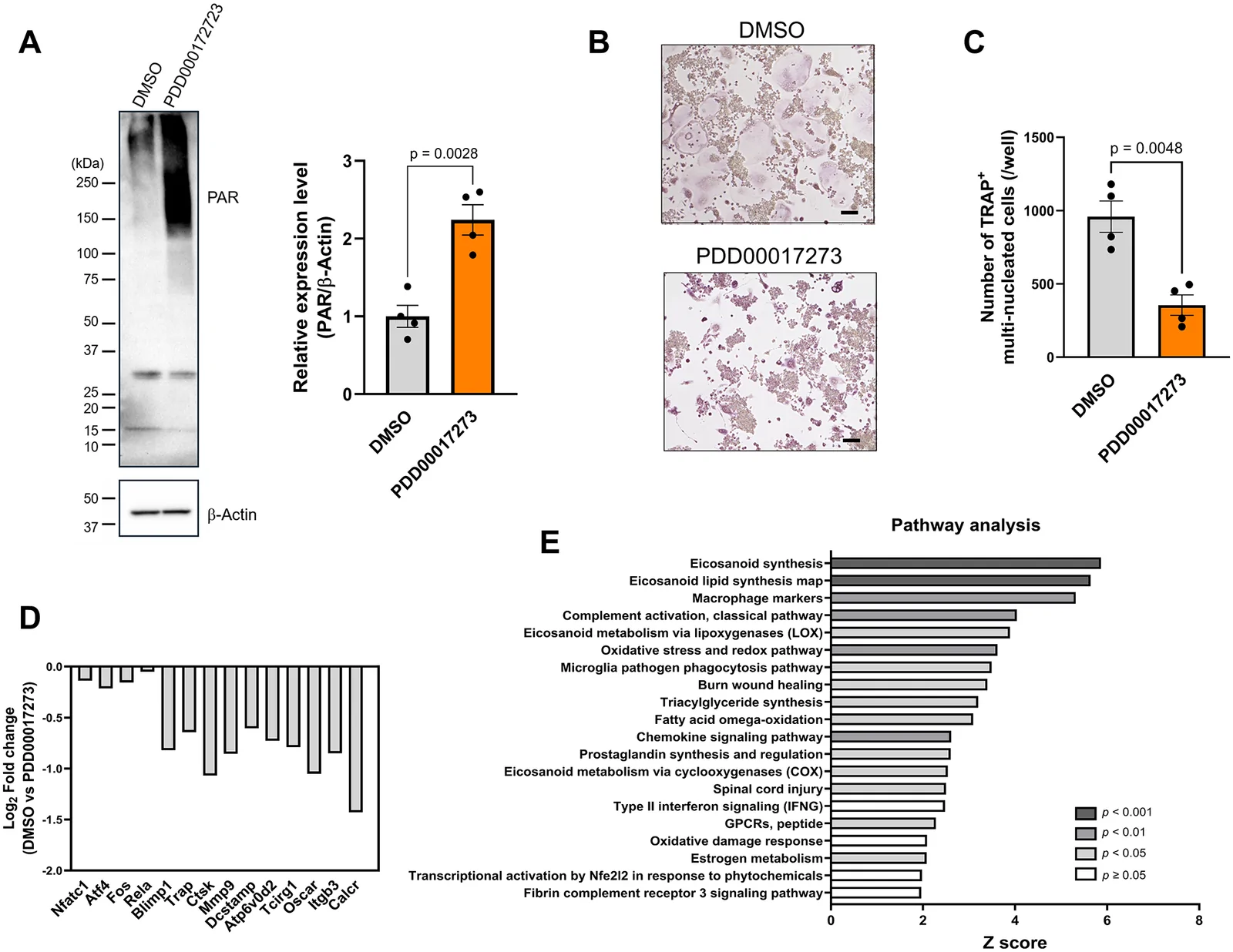
PDD00017273 treatment alters the transcriptional profile and suppresses osteoclast differentiation in RAW264 cells. (A) Intracellular PAR levels in PDD00017273-treated RAW264 cells. RAW264 cells were treated with 1 μM PDD00017273 or DMSO for 2h, and whole-cell extracts were analyzed by western blot analysis using an anti-PAR antibody (left panel). The band intensities were quantified, and the PAR levels were normalized to the β-actin levels (right panel). (B) Images of TRAP staining in DMSO-treated control cells and 1 μM PDD00017273-treated cells at 96 h after RANKL stimulation. Scale bars indicate 100 μm. (C) Quantification of TRAP-positive multinucleated cells (≥3 nuclei per cell) in DMSO-treated control cells and PDD00017273-treated cells. Statistical comparisons were performed using Student’s *t*-test (C). Data are presented as mean ± SEM (n = 4). (D) Changes in expression of osteoclast differentiation-related genes revealed by RNA-seq analysis. (E) A pathway analysis was performed to identify the cellular signaling pathways activated by PDD00017273 treatment compared with control DMSO treatment in RANKL-stimulated cells. DEGs increased by more than 2-fold in PDD00017273-treated cells versus DMSO-treated control cells in the RNA-seq data were used for the pathway analysis. The levels of statistical significance are indicated in the figure.

### PDD00017273 enriches an NRF2-binding motif in accessible chromatin

Next, we investigated how PDD00017273 affects chromatin accessibility and transcriptional regulation in RANKL-stimulated RAW264 cells using ATAC-seq analysis. A correlation analysis based on peak signals in the ATAC-seq data showed high similarity between DMSO-treated control cells and PDD00017273-treated cells (Pearson’s correlation coefficient r = 0.99), suggesting that the overall chromatin accessibility profiles were comparable between the cells. A motif enrichment analysis revealed significant enrichment of a binding motif for the transcription factor Nfe2l2 (Nrf2) (Fig 2A). The ATAC-seq peak patterns at genomic loci of representative NRF2 target genes, including *Hmox1*, *Nqo1*, and *Gclm*, were similar between DMSO-treated control cells and PDD00017273-treated cells (Fig 2B). These findings suggest that PDD00017273 promotes NRF2-dependent transcriptional regulation rather than inducing significant changes in global chromatin accessibility during osteoclast differentiation.

**Fig 2:**
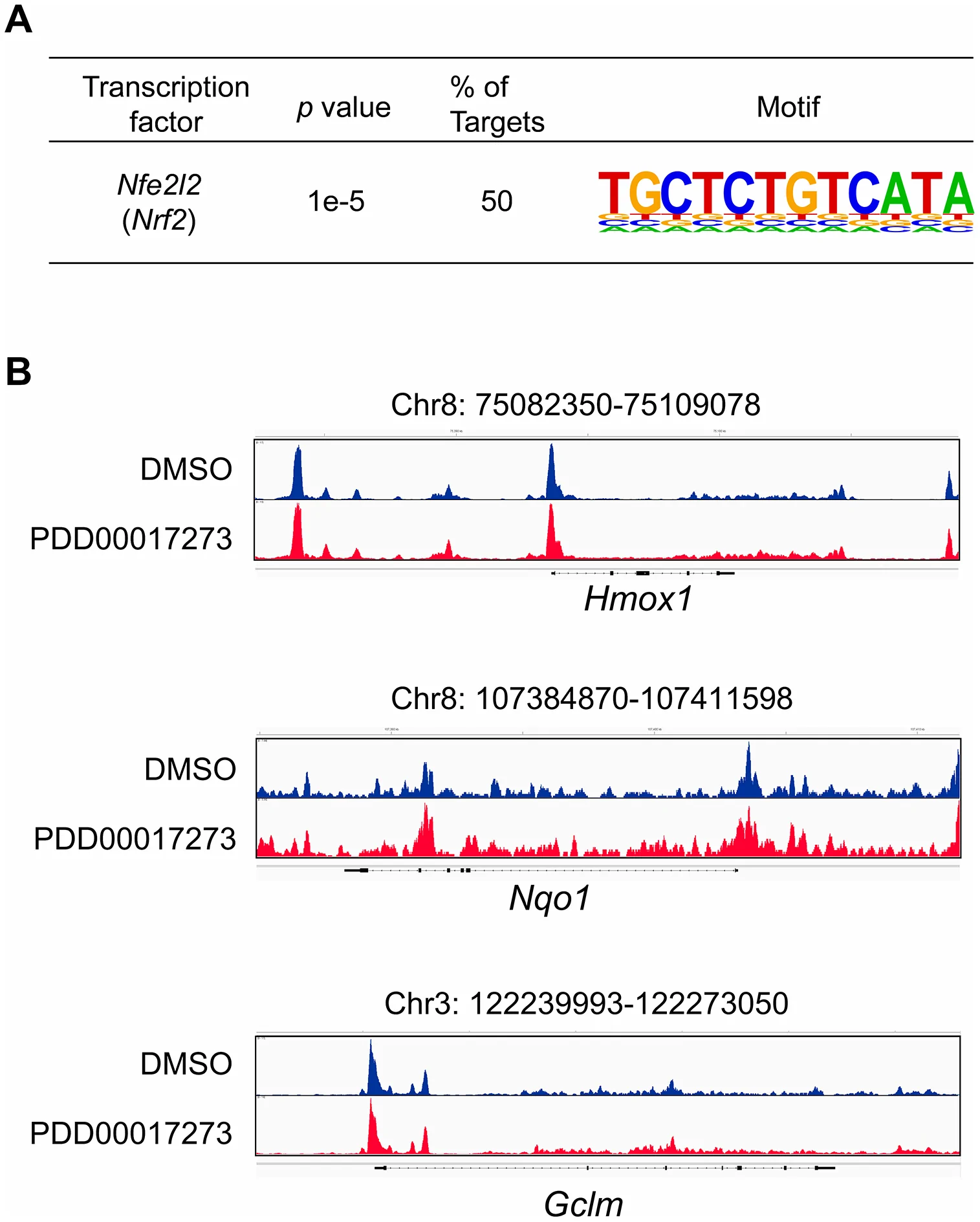
PDD00017273 enriches NRF2-binding motifs without major changes in chromatin accessibility on ATAC-seq analysis. RAW264 cells were treated with 1 μM PDD00017273 in the presence of 200 ng/ml RANKL for 48 h, followed by ATAC-seq analysis. (A) A motif analysis of the accessible chromatin regions identified a binding motif of the transcription factor NRF2. (B) ATAC-seq accessibility coverage tracks for the *Hmox1*, *Nqo1*, and *Gclm* regions, as representative NRF2 target genes, in DMSO-treated control cells and PDD00017273-treated cells.

### PARG inhibition by PDD00017273 induces mRNA expression of NRF2 target genes

To clarify whether PARG inhibition by PDD00017273 enhances NRF2-dependent transcription, we analyzed the mRNA expression levels of antioxidant genes regulated by NRF2 in RANKL-stimulated RAW264 cells treated with PDD00017273. Treatment with PDD00017273 significantly upregulated the expression of NRF2 target genes, including *Hmox1* (heme oxygenase 1), *Nqo1* (NAD(P)H quinone dehydrogenase 1), *Gclm* (glutamate–cysteine ligase modifier subunit), *Slc7a11* (solute carrier family 7 member 11), and *Gsta2* (glutathione S-transferase alpha 2), at 24–72 h after differentiation induction compared with DMSO-treated control cells (Fig 3A–E). In contrast, the expression of *Keap1* (Kelch-like ECH-associated protein 1), a negative regulator of NRF2 [32], was slightly downregulated by PDD00017273 treatment at 48 and 72 h after differentiation induction, while the *Nrf2* mRNA levels were similar to those in DMSO-treated control cells (Fig 3F, G). We therefore analyzed the protein levels of NRF2 and KEAP1 in cytoplasmic and nuclear fractions prepared from the cells at 48 h after differentiation induction using western blot analysis (Fig 3H). NRF2 was detected in the nuclear fraction in both DMSO-treated and PDD00017273-treated cells, and its level did not change markedly after PDD00017273 treatment (Fig 3I). Meanwhile, KEAP1 was detected in the cytoplasmic fraction in both DMSO-treated and PDD00017273-treated cells, and its level also did not change markedly after PDD00017273 treatment (Fig 3J). These results suggest that PDD00017273 may regulate NRF2-dependent transcriptional activity without altering NRF2 protein expression, NRF2 nuclear localization, or KEAP1 protein expression.

**Fig 3:**
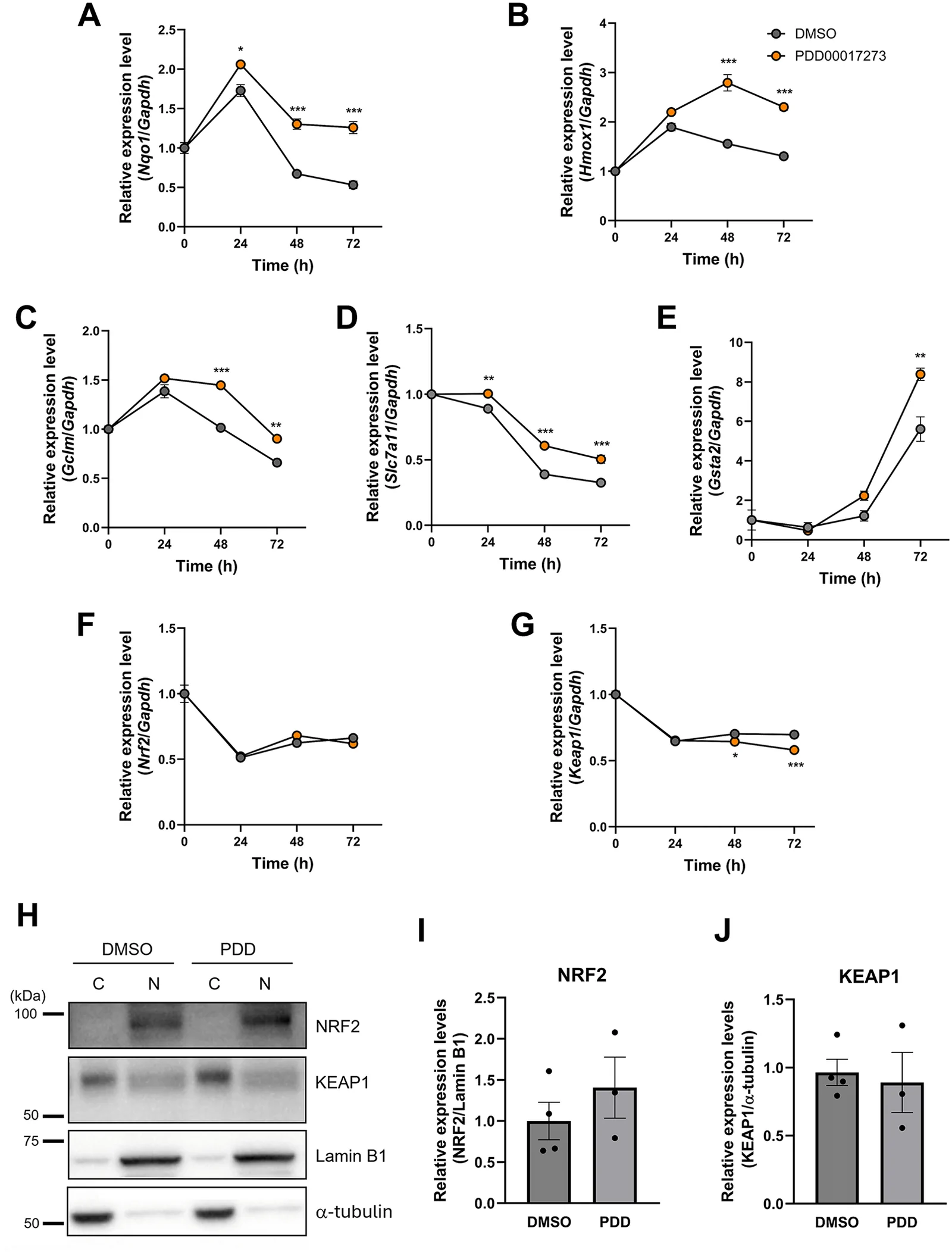
PDD00017273 induces the expression of NRF2 target genes. (A–G) RAW264 cells were treated with DMSO or 1 μM PDD00017273 in the presence of RANKL for 24, 48, and 72 h. The mRNA expression levels of *Hmox1* (A), *Nqo1* (B), *Gclm* (C), *Slc7a11* (D), *Gsta2* (E), *Nrf2* (F), and *Keap1* (G) were analyzed by qRT-PCR. (H) The expression levels of NRF2 and KEAP1 in the cytoplasmic and nuclear fractions were analyzed by western blotting at 48h after differentiation induction in RAW264 cells treated with DMSO or 1 μM PDD00017273 (PDD). C, cytoplasmic fraction; N, nuclear fraction. (I, J) Quantification of NRF2 protein levels in the nuclear fraction (I) and KEAP1 protein levels in the cytoplasmic fraction (J). The band intensities were quantified, and the NRF2 and KEAP1 levels were normalized to the lamin B1 and α-tubulin levels, respectively. No significant differences were observed between DMSO-treated cells and PDD00017273-treated cells. Data are shown as mean ± SEM. *p  <  0.05, **p  <  0.01, and ***p  <  0.001 by Student’s *t*-test.

### Inhibition of NRF2 restores osteoclast differentiation in PDD00017273-treated RAW264 cells

To investigate whether the transcriptional activity of NRF2 affects the suppression of osteoclast differentiation by PARG inhibition, we first examined the effects of different concentrations of the NRF2 inhibitor ML385 on cell viability and TRAP activity. As shown in S1A and S1B Fig, as the ML385 concentration increased, cell viability and the number of TRAP positive multi-nucleated cells decreased concomitantly. Based on these results, we used 2 μM ML385 in subsequent experiments to minimize the effects of ML385 on cell viability and osteoclast differentiation. We then analyzed whether NRF2 inhibition affects the suppression of osteoclast differentiation by PDD00017273. RAW264 cells were treated with 2 μM ML385 in the presence of PDD00017273, and osteoclast formation was evaluated by TRAP activity and TRAP staining. As shown in Fig 4A, co-treatment with ML385 and PDD00017273 partially restored the TRAP activity compared with PDD00017273 treatment alone at 96 h after differentiation induction. Consistent with this finding, co-treatment with ML385 and PDD00017273 significantly increased the number of TRAP-positive multinucleated cells compared with PDD00017273 treatment alone (Fig 4B, C). We also examined the effect of 5 μM ML385 in combination with PDD00017273. Although 5 μM ML385 slightly reduced TRAP activity and the number of TRAP-positive multinucleated cells compared with 2 μM ML385, it similarly attenuated the inhibitory effect of PDD00017273 on osteoclast differentiation (S1C and S1D Fig). These results support the involvement of NRF2 in the suppression of osteoclast differentiation by PDD00017273. Moreover, 2 μM ML385 treatment alone significantly promoted osteoclast formation compared with DMSO control treatment, supporting the notion that NRF2 is a negative regulator of osteoclast differentiation (Fig 4A, B). ML385 treatment alone also significantly increased the TRAP activity and the number of TRAP-positive multinucleated cells compared with DMSO treatment (Fig 4A–C), consistent with a previous report [33]. Moreover, co-treatment with ML385 and PDD00017273 significantly reduced the expression of NRF2-regulated genes, including *Hmox1*, *Nqo1*, *Gclm*, and *Slc7a11*, at 72 h after RANKL stimulation compared with PDD00017273 treatment alone (Fig 5A–D). The expression levels of two osteoclast differentiation markers, *Trap* and *Ctsk*, were also upregulated (Fig 5E, F). These findings suggest that PDD00017273 partially inhibits osteoclast differentiation via activation of NRF2.

**Fig 4:**
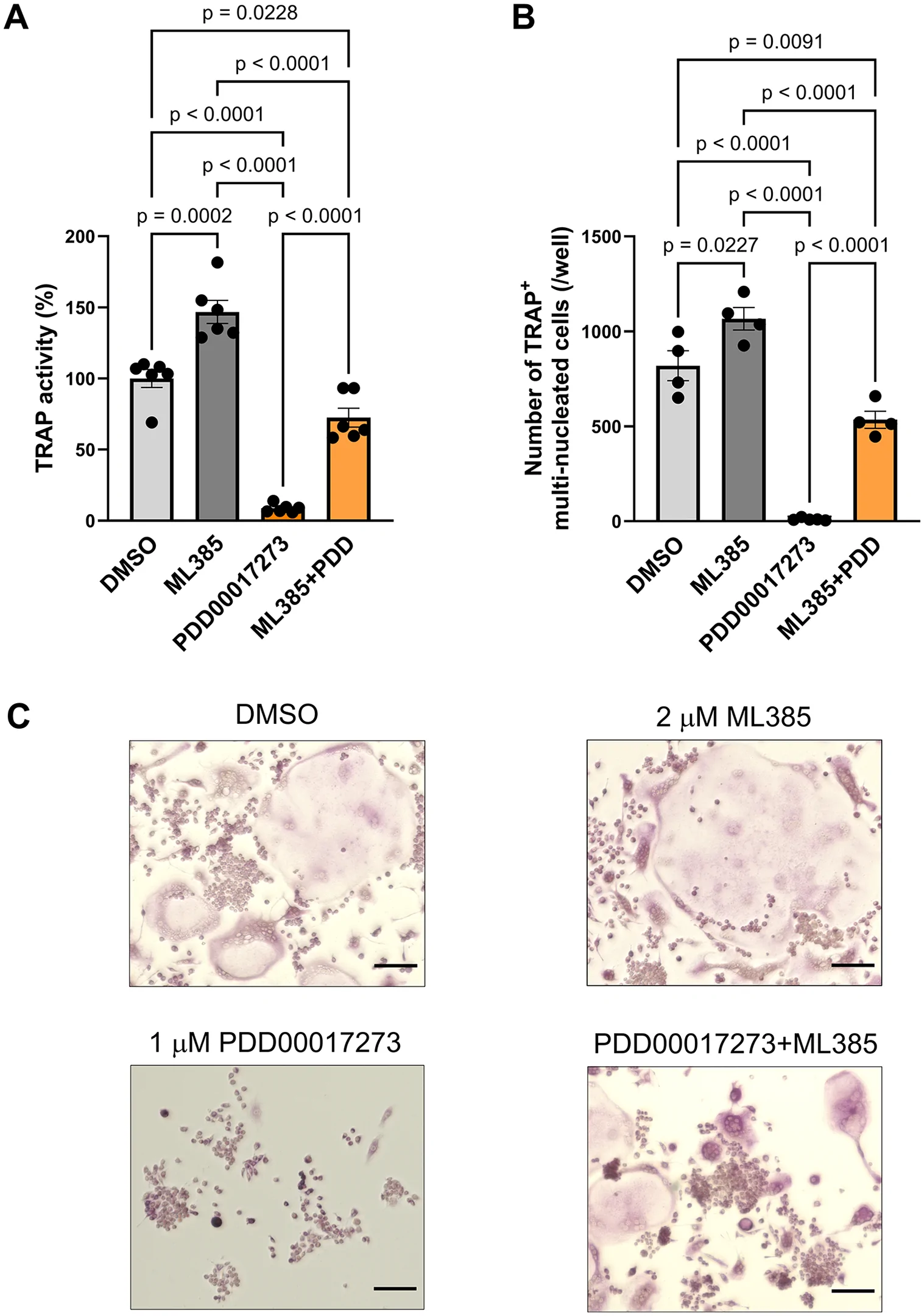
NRF2 inhibition partially restores the suppression of osteoclast differentiation induced by PDD00017273. RAW264 cells were treated with DMSO, 1 μM PDD00017273 (PDD), and/or 2 μM ML385 in the presence of RANKL. (A) TRAP activity was measured at 96 h after differentiation induction. (B) TRAP staining was performed, and the number of TRAP-positive multinucleated cells (≥3 nuclei per cell) was counted. (C) Representative images of TRAP staining in (B). Scale bars indicate 100 μm. ML385 partially restored the osteoclast formation suppressed by PDD00017273. Statistical comparisons were performed using ANOVA, followed by Tukey’s HSD post-hoc analysis (A, B). Data are shown as mean ± SEM (n = 4–6).

**Fig 5:**
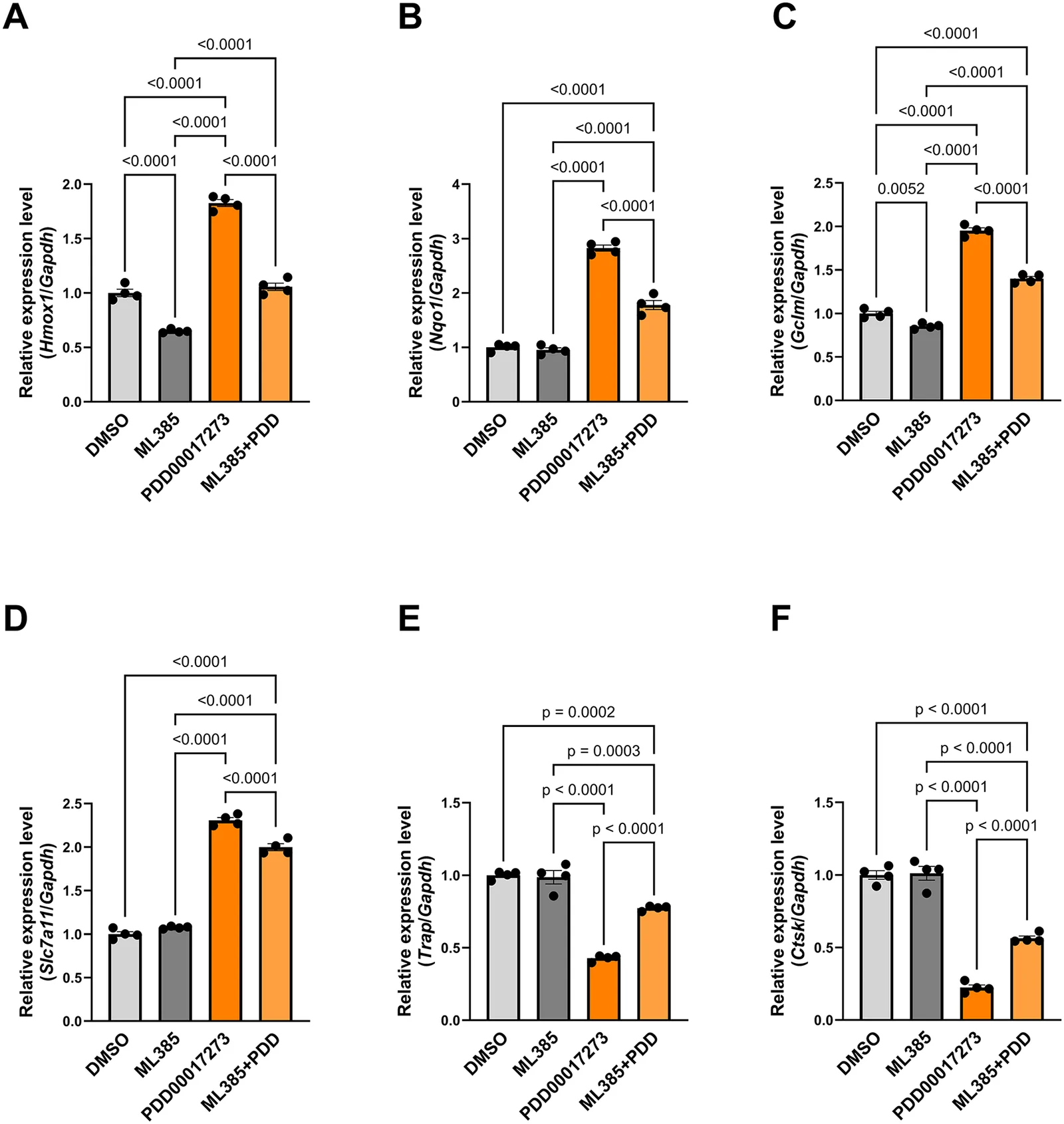
Inhibition of NRF2 suppresses the expression of NRF2 target genes and promotes the expression of osteoclast differentiation markers. RAW264 cells were treated with DMSO, 1 μM PDD00017273 (PDD), and/or 2 μM ML385 in the presence of 200 ng/mL RANKL for 72 h. (A–F) The mRNA expression levels of NRF2 target genes (A, *Hmox1*; B, *Nqo1*; C, *Gclm*; D, *Slc7a11*) and osteoclast differentiation markers (E, *Trap*; F, *Ctsk*) were measured by qRT-PCR. Statistical comparisons were performed using one-way ANOVA, followed by Tukey’s HSD post-hoc analysis. Data are shown as mean ± SEM (n = 4).

## Discussion

Poly ADP-ribosylation is involved in various biological processes, including DNA repair, transcriptional regulation, and chromatin remodeling [18,19]. However, many aspects of the relationship between bone metabolism and PARylation remain unclear, particularly those involving the PARG enzyme. We previously showed that PARG dysfunction through intracellular PAR accumulation suppresses osteoclast differentiation [25], although the molecular mechanisms underlying this phenomenon were not determined. In the present study, we found that NRF2 activation is involved in the suppression of osteoclast differentiation under PARG inhibition. These findings suggest that altered PAR metabolism suppresses osteoclast differentiation through an NRF2-mediated mechanism.

Increased PARylation has been reported to induce chromatin relaxation. Briefly, PARylation of PARP1 itself and histones can change the chromatin structure and facilitate the recruitment of PAR-dependent chromatin remodelers, such as ALC1, APLF, and CHD2 [34,35]. In the present study, ATAC-seq analysis revealed that chromatin accessibility was largely unchanged between DMSO-treated control cells and PDD00017273-treated cells. These findings indicate that the suppression of osteoclast differentiation induced by PDD00017273 is not associated with dynamic changes in the chromatin structure. Instead, our findings support a central role for transcriptional regulation, particularly that PAR accumulation or PARylation of specific proteins may promote NRF2-dependent transcription without dramatic changes in the chromatin structure.

Under normal conditions, NRF2 is localized in the cytoplasm as a complex with KEAP1, which mediates its degradation [32]. However, under oxidative stress, NRF2 becomes activated and translocates to the nucleus [36], where it promotes the transcription of target genes [37,38]. Notably, NRF2 is known as a negative regulator of osteoclast differentiation. Briefly, deletion of NRF2 was shown to promote osteoclast differentiation in bone marrow-derived cells and RAW264.7 cells, accompanied by activation of NFATc1, a master regulator of osteoclast differentiation [33,39]. Overexpression of NRF2 was found to inhibit osteoclast differentiation both in vitro and in vivo through the upregulation of antioxidant genes, such as *Hmox1* [33,40]. As previously reported, PARP family proteins are involved in the regulation of NRF2 activity. PARP2 has been reported to promote the nuclear localization of NRF2 through PARylation in C2C12 cells [41]. In addition, PARP1 interacts with NRF2 and promotes the transcriptional activity of NRF2 [42]. In this study, PARG inhibition by PDD00017273 induced the upregulation of NRF2-regulated genes, including *Hmox1* and *Nqo1*. Furthermore, pharmacological inhibition of NRF2 promoted osteoclast formation in PDD00017273-treated cells. However, PDD00017273 treatment did not induce any changes in NRF2 protein expression or subcellular localization. Similarly, KEAP1 protein expression remained unchanged after PDD00017273 treatment. These findings indicate that the increased NRF2-dependent transcriptional activity cannot be readily explained by changes in NRF2 protein expression or nuclear localization, or in cytoplasmic KEAP1 protein expression. Taken together, these findings suggest that PARG inhibition may regulate NRF2 transcriptional activity through PARylation of target proteins, including NRF2 itself. Furthermore, the RNA-seq analysis showed that the expression levels of *Parp1* and *Parp2* did not differ significantly between DMSO-treated control cells and PDD00017273-treated cells, suggesting that PAR accumulation mainly arises through PARG inhibition rather than through increased PAR synthesis by PARP enzymes. Based on these findings, possible mechanisms may include transcriptional activation of NRF2 through PARylation of NRF2 itself and indirect effects of PAR accumulation on the KEAP1-NRF2 pathway, which may ultimately influence NFATc1 activation and osteoclast differentiation. Further research is needed to elucidate the relationship between PARylation and the KEAP1-NRF2 pathway. In addition to PARG, other PAR-degrading enzymes may also contribute to the regulation of PAR metabolism during osteoclast differentiation. ADP-ribosyl-acceptor hydrolase 3 (ARH3) catalyzes exoglycosidic degradation of PAR [43,44] and therefore may contribute to the regulation of intracellular PAR levels. Furthermore, ARH3 possesses mono-ADP-ribose hydrolase activity toward Ser-ADP-ribose, thereby contributing to the regulation of ADP-ribosylation [45]. Thus, elucidation of the contribution of ARH3 to the regulation of PAR metabolism and osteoclast differentiation remains an interesting subject for future investigations.

Previous studies have indicated that NRF2 negatively regulates osteoblast differentiation. Specifically, overexpression of NRF2 was shown to suppress osteoblast differentiation by inhibiting the RUNX2-dependent transcriptional activity in pre-osteoblastic MC3T3-E1 cells [46]. NRF2 knockout mice were observed to exhibit increased bone mass compared with wild-type mice through enhanced bone-forming activity of osteoblasts [47]. In the present study, PDD00017273 suppressed osteoclast differentiation through NRF2 activation. Moreover, we previously demonstrated that PDD00017273 accelerates osteoblast differentiation in pre-osteoblastic MC3T3-E1 cells [48]. Thus, PARG dysfunction may regulate osteoclast and osteoblast differentiation through NRF2-mediated transcriptional regulation, while the precise mechanisms remain unclear. In the bone microenvironment, osteoclasts and osteoblasts cooperate to regulate bone remodeling [1]. PARG inhibition by PDD00017273 could exert a dual effect by suppressing osteoclast differentiation and promoting osteoblast differentiation via NRF2 activation, which may assist in the maintenance of bone homeostasis. Abnormalities in bone remodeling are involved in the progression of metabolic bone diseases, such as osteoporosis. Therefore, regulation of PARG activity may be a potential therapeutic strategy to suppress bone resorption while promoting bone formation. Taken together, our findings suggest that PARG could be a therapeutic target for metabolic bone diseases by inhibiting osteoclast differentiation via NRF2-dependent mechanisms.

## Supporting information

S1 Fig Effects of different concentration of ML385 on cell viability and TRAP activity and attenuation of PDD00017273-induced suppression of osteoclast differentiation by 5 μM ML385. (A) RAW264 cells were cultured in the presence of 0–20 µM ML385 for 3 days. Cell viability was measured by the CCK-8 assay. (B) RAW264 cells were treated with 0–20 μM ML385 in the presence of 200 ng/mL RANKL for 72h. Osteoclast differentiation was evaluated by TRAP activity. (C, D) RAW264 cells were treated with 5 μM ML385 and/or 1 μM PDD00017273 (PDD) for 96h, and osteoclast differentiation was evaluated by TRAP activity (C) and TRAP staining (D). Representative images of TRAP-stained cells after treatment with ML385 alone or both ML385 and PDD00017273 are shown in the right panel. The DMSO-treated and PDD00017273-treated cells in (D) are the same as those shown in Fig 4B. Statistical comparisons were performed using one-way ANOVA, followed by Tukey’s HSD post-hoc analysis. Data are shown as mean ± SEM (n = 4–6).
(PDF)
