# Supplementary material for: PARG inhibition suppresses osteoclast differentiation through transcriptional activation of an NRF2-dependent pathway
Source: PLoS One. 2026 Oct 26;21(10):e0361101. doi: 10.1371/journal.pone.0361101 (PMC13704809; doi:10.1371/journal.pone.0361101)
Supplement: Supplementary file 1 [file pone.0361101.s001.pdf]

S1 Fig

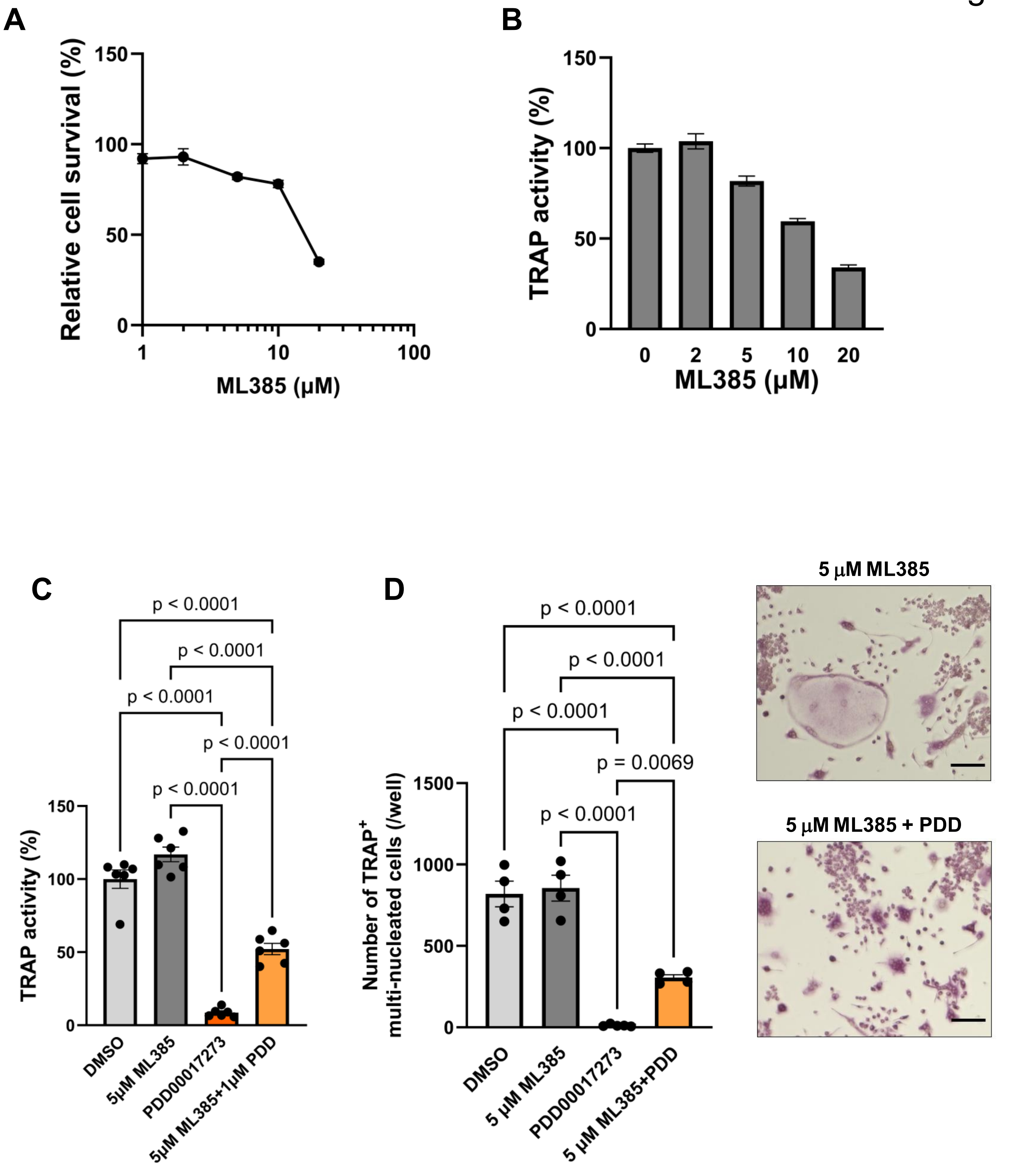

A
Relative cell survival (%)
150
100
50
0
1
10
100
ML385 (μM)
B
TRAP activity (%)
150
100
50
0
0
2
5
10
20
ML385 (μM)
C
p < 0.0001
p < 0.0001
p < 0.0001
p < 0.0001
p < 0.0001
TRAP activity (%)
150
100
50
0
DMSO
5μM ML385
PDD00017273
5μM ML385+1μM PDD
D
p < 0.0001
p < 0.0001
p < 0.0001
p = 0.0069
p < 0.0001
Number of TRAP+ multi-nucleated cells (/well)
1500
1000
500
0
DMSO
5 μM ML385
PDD00017273
5 μM ML385+PDD
5 μM ML385
5 μM ML385 + PDD
